# Use of Transnational Services to Prevent Treatment Interruption in Tuberculosis-Infected Persons Who Leave the United States

**DOI:** 10.3201/eid2203.141971

**Published:** 2016-03

**Authors:** Cynthia A. Tschampl, Deborah W. Garnick, Edward Zuroweste, Moaven Razavi, Donald S. Shepard

**Affiliations:** Brandeis University, Waltham, Massachusetts, USA (C.A. Tschampl, D.W. Garnick, M. Razavi, D.S. Shepard);; Migrant Clinicians Network, Austin, Texas, USA (E. Zuroweste)

**Keywords:** continuity of patient care, treatment interruption, emigration, immigration, incidence, multidrug-resistant tuberculosis, tuberculosis, TB, multidrug resistance, drug resistance, United States, cross-border cases, antimicrobial resistance, tuberculosis and other mycobacteria, interventions

## Abstract

A major problem resulting from interrupted tuberculosis (TB) treatment is the development of drug-resistant TB, including multidrug-resistant TB (MDR TB), a more deadly and costly-to-treat form of the disease. Global health systems are not equipped to diagnose and treat the current burden of MDR TB. TB-infected foreign visitors and temporary US residents who leave the country during treatment can experience treatment interruption and, thus, are at greater risk for drug-resistant TB. Using epidemiologic and demographic data, we estimated TB incidence among this group, as well as the proportion of patients referred to transnational care–continuity and management services during relocation; each year, ≈2,827 visitors and temporary residents are at risk for TB treatment interruption, 222 (8%) of whom are referred for transnational services. Scale up of transnational services for persons at high risk for treatment interruption is possible and encouraged because of potential health gains and reductions in healthcare costs for the United States and receiving countries.

## Abstract 2

Scale up of such services is possible and encouraged because of potential health gains and reduced healthcare costs.

Drug-resistant tuberculosis causes tremendous suffering and high death rates, as well as disruption to public health budgets and TB control efforts (*1*,*2*). Multidrug-resistant TB (MDR TB), defined as TB resistant to the 2 main TB drugs, is a growing concern, and current global health systems are inadequate to deal with this airborne, deadly pandemic disease (*3*,*4*). Mobile populations are more likely to have TB because of various risk factors (e.g., crowded housing and stress of relocating) and to spread TB in the absence of timely and effective intervention (*5*,*6*). Most TB cases in high-income nations are in persons born outside those nations (*7*,*8*). Mobility also contributes to a risk for treatment interruption, a key cause of drug resistance (*5*,*6*,*9*). 

An understanding of the magnitude and dynamics of treatment interruption among mobile populations is essential for public health surveillance and policymaking. To elucidate this problem, we used epidemiologic and demographic data from organizations such as the World Health Organization (WHO), US Department of Homeland Security (DHS), and Pew Hispanic Center to estimate the incidence of TB in a population at elevated risk for drug resistance, namely foreign-born persons who depart the United States before clinically recommended TB treatment was completed. We then estimated the proportion of those persons who received transnational care–continuity services by using case management data from the provider organizations (the nonprofit Migrant Clinicians Network [MCN], Austin, Texas, USA, and the County of San Diego TB Control Program, San Diego, CA, USA).

## Methods

### Population

The study population included any nonimmigrant, nonrefugee, nonnative visitor to the United States during 2008–2012 who had TB and left the country before treatment completion (Table 1). Because persons visit the United States from many countries and via many routes, both legal and illegal, the study population was categorized into subgroups. Sufficient data were available to calculate person-years among those temporarily in the United States with authorization. This subpopulation included all nonimmigrant visitors and temporary residents because they had been in the country long enough to receive a diagnosis of TB but had visa restrictions that nearly assured TB treatment would not be finished before they left. 

**Table 1:** Study population inclusion and exclusion criteria, data sources, and estimation equations used to determine number at risk of treatment interruption among TB-infected, authorized and unauthorized visitors to the United States, 2008-2012*

Subgroup	Justification	References	Calculation method	
Included in study				
Resided in the United States with authorization†				
Tourist or business travelers	Left United States after <2 mo	(*10*–*15*)	PY × (country-specific TB incidence rate)‡	
Student or exchange visitors	Left United States after <9 mo	(*10*–*15*)	PY × (country-specific TB incidence rate)‡	
Temporary workers	Left United States after <5 mo	(*10*–*15*)	PY × (country-specific TB incidence rate)‡	
Diplomat or other representatives	Left United States after <3 mo	(*10*–*15*)	PY × (country-specific TB incidence rate)‡	
Persons with all other visa types	Left United States after <1 mo	(*10*–*15*)	PY × (country-specific TB incidence rate)‡	
Persons with unknown visa type	Left United States after <2 mo	(*10*–*15*)	PY × (country-specific TB incidence rate)‡	
Visitors from Canada and MX without I-94 card	Left United States after <1 mo	(*10*–*15*)	PY × (country-specific TB incidence rate)‡	
Resided in United States without authorization			
Detained first and then removed by US officials	Left United States; subgroup in this category for which most data was available	(*12*,*16*,*17*)	No. detainees × (183/365) × TB incidence rate for detainees × proportion removed§	
All other removals meeting inclusion criteria	Left United States	(*10*,*12*,*16*,*17*)	(No. nonexpedited removals × estimated no. detained before removal) × (183/365) × (country-specific TB incidence rate)¶	
MX nationals leaving United States of own volition	Left United States	(*10*,*12*,*16*,*18*,*19*)	No. MX nonexpedited removals × estimated % left voluntarily × (183/365) × (MX TB incidence rate)#	
All other nationals leaving United States of own volition	Left United States	(*10*,*12*,*16*,*18*,*19*)	(Total who left voluntarily − MX left voluntarily) × (183/365) × (57/100,000 PY)**	
Excluded from study				
Resided in the United States with authorization				
Immigrants	Permanent residents; no requirement to leave United States	(*12*–*15*)	NA	
Refugees	Permanent residents	(*12*–*15*)	NA	
Asylees	Permanent residents	(*12*–*15*)	NA	
Resided in the United States without authorization				
Currently residing in the United States	Did not leave United States during study period	(*12*,*16*,*18*)	NA	
Returnees and expedited removals††	Did not officially enter United States	(*12*,*16*,*18*)	NA	
Detained but not removed	Did not leave United States during study period	(*12*,*16*,*18*)	NA	

*Study population is defined as those who were born outside the United States, had active tuberculosis while in the United States, and then left the United States before treatment completion was possible. I-94 card, the entry/exit form that all nonimmigrant visitors (except certain ones from MX and Canada) must fill out; MX, Mexico/Mexican; NA, not applicable; PY, person-years; TB, tuberculosis. †These subgroups included family members. See Technical Appendix Table 1 for a complete list of visas for each subgroup and their corresponding mean and median length of stay. ‡Calculated for all countries, 2008–2012. PY = no. of admissions × (weighted mean length of stay in days/365). Weighted median length of stay was used for all these groups in sensitivity analyses, except those without an I-94 card, as only the mean was available. See Technical Appendix Tables 3–6 for results. §183 d, or 6 mo, of risk was assumed as the minimum amount of time for TB to be diagnosed, treatment started, and a treatment interruption caused by leaving the United States. ¶Calculated for top 12 receiving countries by using World Health Organization country-specific TB incidence rates. All other countries grouped together and multiplied by the midpoint TB incidence rate of 57 cases/100,000 PY. #Calculated for MX nationals; they make up the majority (assumed at 90%) of this subgroup. **All other countries’ nationals assumed to make up 10% of this subgroup; the midpoint incidence rate of TB burden was 57 cases/100,000 PY. ††These are 2 immigration enforcement categories with specific definitions used by US Department of Homeland Security (*16*).

We classified authorized visitors into 7 categories (Technical Appendix Table 1): tourists and business travelers, students and exchange visitors, temporary workers, diplomats and other representatives, persons with any other visa class, persons with unknown visa class, and Canada and Mexico nationals not requiring an entry–exit (I-94) card. The remaining persons within the study population were in the country without authorization and were divided into 4 data-driven groupings: persons detained and then removed by US officials (nonexpedited), all other nonexpedited removals, persons from Mexico who voluntarily left, and all other persons who voluntarily left. 

Six subgroups, including an expedited removal subgroup, were excluded (Table 1). Exclusion criteria comprised permanent US residency and no US entry or exit during the study period. MCN and Brandeis University (Waltham, Massachusetts, USA) Institutional Review Boards approved this study.

### Data

To estimate incident TB cases, we needed TB incidence rates and number of person-years for each subgroup. We obtained person-years by combining an appropriate measure of time at risk for active TB with a measure of magnitude (e.g., number of nonimmigrant visa admissions) (Table 2). We obtained country-specific TB incidence rates per 100,000 person-years from WHO (*10*). As in other studies (*11*), we defined countries with high, medium, and low TB incidence as >100, 15–99, and 0–14 cases per 100,000 person-years, respectively.

**Table 2:** Admissions, person-years, incident tuberculosis cases, and case rates stratified by visa group and tuberculosis burden level for persons temporarily in the United States, with authorization, 2008–2012*

Visa group†	Admissions (%)	PY (%)	Tuberculosis		
			Total no. cases (%)	No. cases/100,000 PY (95% CI)	No. cases/100,000 admissions (95% CI)
Tourist and business traveler	201,578,207 (25)	14,431,062 (47)	6,161 (48)	43 (36–49)	3 (3–4)
High-burden countries	13,858,503 (2)	1,277,466 (4)	2,614 (20)	205 (174–235)	19 (16–22)
Medium-burden countries	126,042,138 (15)	10,733,970 (35)	3,342 (26)	31 (26–36)	3 (2–3)
Low-burden countries	61,677,566 (8)	2,419,625 (8)	205 (2)	8 (7–10)	0
Student/exchange visitor‡	9,417,888 (1)	6,293,260 (21)	3,675 (28)	58 (50–67)	39 (33–45)
High-burden countries	1,862,032	1,244,255 (4)	2,040 (16)	164 (139–189)	110 (93–126)
Medium-burden countries	4,932,913 (1)	3,296,292 (11)	1,516 (12)	46 (39–53)	31 (26–35)
Low-burden countries	2,622,943	1,752,714 (6)	118 (1)	6 (5–7)	5 (4–5)
Temporary worker‡	12,904,847 (2)	4,948,262 (16)	2,319 (18)	47 (40–54)	18 (15–21)
High-burden countries	2,154,566	826,151 (3)	1,604 (12)	194 (165–223)	74 (63–86)
Medium-burden countries	5,252,984 (1)	2,014,215 (7)	587 (5)	29 (25–34)	11 (10–13)
Low-burden countries	5,497,297 (1)	2,107,895 (7)	128 (1)	6 (5–7)	2 (2–3)
Diplomat and other representative‡	1,761,901	381,343 (1)	243 (2)	64 (54–73)	14 (12–16)
High-burden countries	332,182	71,897	167 (1)	232 (198–267)	50 (43–58)
Medium-burden countries	819,393	177,348 (1)	66 (1)	37 (31–42)	8 (7–9)
Low-burden countries	610,326	132,098	10	8 (7–9)	2 (1–2)
All other classes	2,267,465	119,836	107 (1)	90 (76–103)	5 (4–5)
High-burden countries	905,522	38,206	89 (1)	232 (197–267)	10 (8–11)
Medium-burden countries	1,107,955	46,747	18	38 (32–44)	2 (1–2)
Low-burden countries	253,988	34,884	0.8	2 (2–3)	0
Unknown visa class	1,123,438	90,579	52	57 (49–66)	5 (4–5)
High-burden countries	71,316	6,643	16	236 (200–271)	22 (19–25)
Medium-burden countries	792,676	73,838	35	47 (40–54)	4 (4–5)
Low-burden countries	259,446	10,098	2	17 (14–20)	1 (1–1)
Canada and Mexico nonimmigrant without I-94 card	592,645,430 (72)	4,266,235 (14)	371 (3)	9 (7–10)	0
Total	821,699,176	30,530,577	12,928	NA	NA
Annual average	164,339,835	6,106,115	2,586	NA	NA


*I-94 card, the entry/exit form that all nonimmigrant visitors (except certain ones from Mexico and Canada) must fill out; NA, not applicable; PY, person-years. US, United States. †High-burden countries were defined as having >100 TB incident cases/100,000 PY; medium-burden countries were defined as having 15–99 cases/100,000 PY, and low-burden countries were defined as having 0–14 cases/100,000 PY. ‡Corresponding spouses and children are also included in each of these categories; see Technical Appendix Table 1 for full list of visas included in each subgroup.

We obtained the number of nonimmigrant visas issued in 2008–2012 from the US Department of State (*15*) and the number of nonimmigrant visa admissions with median and mean lengths of stay (LOS) for each country from DHS (*12*,*13*). We categorized nonimmigrant visa admissions into 7 groups, including a group of nonimmigrant visitors from Canada and Mexico without an I-94 card. DHS also provided data on the proportion of these admissions from Canada (28.5%) and Mexico (71.5%) (*12*,*14*).

We used DHS data (reported in aggregate and categorized by top receiving countries) on the number of compulsory and confirmed departures from the United States (*12*,*16*). To extrapolate the number of voluntary exits for persons from Mexico, we used previously estimated percentages (*18*) of Mexican nationals involuntarily returning home and mean LOS before removal. We used data reported by Schneider and Lobato (*17*) on TB case rates and removal rates for persons detained by US immigration officials.

We estimated the number of persons served by transnational care coordination services by using published case management data from the 2 existing referral programs, Health Network (previously known as TBNet) and CureTB. MCN operates Health Network, which began in 1998 and provides bridge case management, care continuity, patient education and navigation, and bidirectional communication between providers on behalf of patients for high-value interventions. In 2011, Health Network managed patients returning to >50 countries and achieved an 84.7% treatment completion rate (*20*). CureTB, operated by the County of San Diego, started managing binational (United States and Mexico) TB cases in 1997 and recently expanded to manage cases in persons moving to Central America; CureTB reported a 79% treatment completion rate (*21*). 

### Statistical Analysis

Some subgroups had better data available for estimating incident TB cases; therefore, we present the analyses in order of increasing complexity (Table 1) and then discuss calculations regarding the transnational care–continuity services. First, we estimated incident TB cases for authorized visitors in the United States stratified by visa group, country, and year and subsequently aggregated across levels of TB burden before final summation. We started by calculating a weighted mean LOS for each visa group (Technical Appendix Table 1) and then applied the following equation (Equation 1): 

incident TB cases = (person-years for visitors with authorization) × (country-specific TB incidence)

where person-years = (no. of admissions) × (mean LOS in days/365 days per year). For example, in 2008 there were 163,845 persons from South Korea in the students and exchange visitors subgroup who stayed a mean of 244 days, resulting in 109,485 person-years (*12*). As a sensitivity analysis, we substituted available weighted median LOS and found 79,005 person-years (Technical Appendix).

For the group with unknown visa type, we used mean LOS (34 days) for all visas (*13*). Persons from Canada and Mexico without an I-94 card had a mean LOS of 3.7 and 1.1 days, respectively (*13*). We used birth-country TB case rates because past studies suggested TB activation rates among non–US-born persons most closely match their TB risk at home (*22**,**23*). For admissions with no country, we applied the midpoint rate from the medium-incidence category (i.e., 57 cases/100,000 person-years) after testing it against the global average rate of 122 cases/100,000 person-years (*24*).

We further calculated TB cases per 100,000 person-years and 100,000 admissions, along with 95% CIs, assuming a Poisson distribution (Technical Appendix). Another sensitivity analysis, using I-94 visa counts from US Department of State (*15*), provided an alternative to the 95% CI. We calculated the range within which the actual number of cases should fall by adapting equation 1. For the lower bound, we assumed 1 admission per visa (despite multiple-entry visas) and replaced admissions with visa counts. For the upper bound, we assumed each visitor had 12 months of risk, the highest possible value.

Second, we estimated TB cases for persons in the United States without authorization whom US officials removed. We began by adapting Equation 1 and multiplying by proportion (*17*) of persons removed postdetention (Table 1). We assumed a 6-month risk for all unauthorized subgroups because that is the minimum amount of time required to receive a diagnosis of TB infection, begin treatment, and still leave the United States before treatment completion. Sensitivity analyses included varied parameters of time at risk, TB case rate, and proportion removed (Technical Appendix). We then estimated, again adapting Equation 1, TB cases for all remaining persons who were in the country without authorization. For these person-years, we separately calculated removals for each year among the group of top receiving countries (i.e., Brazil, China, Colombia, Dominican Republic, Ecuador, El Salvador, Guatemala, Honduras, India, Jamaica, Mexico, Nicaragua) and among the all other countries group. For the all other countries group, we used the midpoint TB case rate (57 cases/100,000 person years). In sensitivity analyses, we varied the time at risk for TB from a maximum of 9 months to a minimum represented by a weighted mean LOS in the United States before removal (i.e., 140 days) (*18*). This calculation was done for all 4 subgroups of persons in the United States without authorization.

Third, we estimated TB cases for persons in the United States without authorization who subsequently voluntarily left. Because most of this subpopulation consists of persons from Mexico, which is also the group for which most data were available (*18*), we began with the DHS-reported numbers of total nonexpedited removals of Mexican nationals (*16*). We applied equation 1 to the following unique person-years (Equation 2): 

person-years of unauthorized Mexican nationals leaving US on own = ([total unauthorized Mexican nationals leaving] − [Mexican, nonexpedited removals]) × (183/365)

where total unauthorized Mexican nationals leaving = Mexican nonexpedited removals/35%. We used the highest proportion of involuntary to voluntary departures (35:65) (*18*) because of an increase in removals in the past decade (*25*). A report from Mexico on migratory flows provided corroborative evidence for our estimate of total departures of Mexican nationals (*19*).

To obtain the final estimate of TB cases among subgroups without authorization, we assumed that persons from Mexico made up 90% of those who voluntarily left the United States because they are the documented majority of migrants (*18*), Mexico is a bordering nation, and local antiimmigration laws tend to target unauthorized visitors from Mexico (*19*,*26*). We then adapted Equation 2 and applied the 90% assumption.

Next, we estimated the number in the study population who were referred for transnational care–continuity services by extrapolating from and adding previously reported provider data (*20**,**21*,*27*,*28*). No evidence was found that any of these persons met 1 of 4 relevant exclusion criteria.

Last, we calculated the proportion of the study population who received transnational services to mitigate drug resistance and other negative consequences of interrupted TB treatment. To do this, we divided the number of persons receiving services by the estimated number of incident TB cases. We also estimated the proportion of referred cases included in the detained-then-removed subgroup. 

## Results

The cumulative number of incident TB cases among the study population was 14,134, and the annual average incidence was 2,827 cases (95% CI 2,440–3,213; Table 3) among an estimated annual population of 6.9 million. The sensitivity analysis using available median LOS resulted in 1,544 annual cases (95% CI 1,249–1,840; Technical Appendix Tables 3–6). Further sensitivity analysis using visa count data produced an annual range of 1,352–4,637 cases.

**Table 3:** Estimated number of incident tuberculosis cases for all subgroups at risk for treatment interruption due to voluntary or involuntary departure from the United States, 2008–2012*

**Table 3.** Estimated number of incident tuberculosis cases for all subgroups at risk for treatment interruption due to voluntary or involuntary departure from the United States, 2008–2012*						
Study subgroup	No. cases, by year					Yearly average (%)
	2008	2009	2010	2011	2012	
Resided in United States with authorization						
Tourist and business traveler	1,099	987	1,219	1,403	1,454	1,232 (44)
Student and exchange visitor†	696	657	785	791	745	735 (26)
Temporary worker†	474	394	473	503	475	464 (16)
Diplomat and other representative†	47	46	50	50	49	49 (2)
All other NIV classes	24	22	21	21	20	21 (1)
Unknown NIV class	10	9	15	10	8	10
Canada residents, no I-94 card	21	19	15	15	15	17 (1)
Mexico residents, no I-94 card	64	60	54	52	55	57 (2)
Resided in United States without authorization						
Detained then removed	173	175	166	196	218	186 (7)
Nondetained, removed	6	6	6	6	6	6
Mexico resident, voluntary departures	35	42	39	40	39	39 (1)
All other voluntary departures	10	12	11	11	11	11
Total	2,659	2,430	2,853	3,099	3,094	2,827

*I-94 card, the entry/exit form that all nonimmigrant visitors (except certain ones from Mexico and Canada) must fill out; NIV, nonimmigrant visa. †Corresponding spouses and children were included in each of these categories; see Technical Appendix Table 1 for full list of visas included in each subgroup.

For the authorized subpopulations, we calculated a total of 30,530,577 person-years and 12,928 cases during 2008–2012. Tourist and business travelers represented 47% (14,431,062) of these person-years; students and exchange visitors, 21% (6,293,260); temporary workers, 16% (4,948,262); diplomats, 1.2% (381,343); and persons from Canada and Mexico without an I-94 card 14% (4,266,235). Tourist and business travelers from medium-incidence countries accounted for most cases (3,342; 26%). However, students and exchange visitors from countries with a high TB incidence had the highest number of cases per 100,000 admissions (110, 95% CI 93–126), followed by temporary workers from high-incidence countries (74, 95% CI 63–86), diplomats from high-incidence countries (50, 95% CI 43–58), and students and exchange visitors from medium-incidence countries (31, 95% CI 26–35).

Among the subpopulations without authorization, we calculated a total of 1,206 incident TB cases, representing an annual average of 241 (Table 3). Persons removed by US officials and those who left voluntarily represented 958 and 259 cases, respectively. These subpopulations represented 8.5% (241/2,827) of annual cases (Figure). Sensitivity analyses showed an annual range of 180–324 cases (6.4%–11.5% of total).

**Figure:**
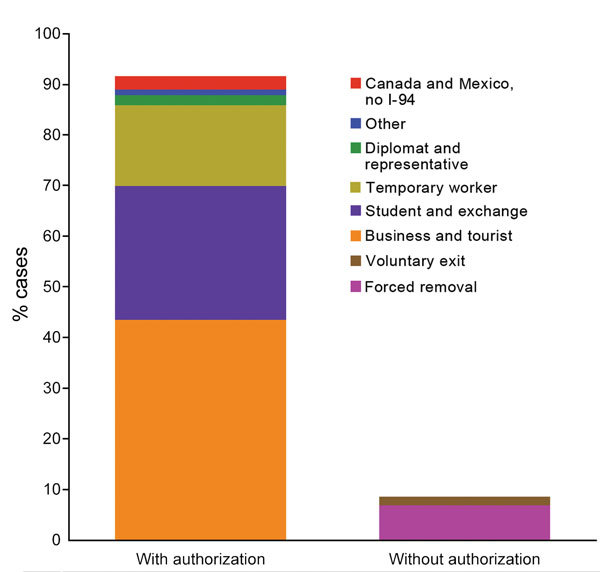
Estimated percentage of incident tuberculosis cases among authorized and unauthorized visitors to the United States who were at risk for treatment interruption due to voluntary or involuntary departure from the country, 2008–2012. Key indicates subgroups of visitors by visa status.

We estimated CureTB and Health Network managed 510 and 599 TB cases, respectively, for a collective annual average of 222 cases during the study period (Table 4). Thus, 7.9% (222/2,827) of persons leaving the United States before treatment completion received transnational care–continuity services. We further estimated that 67% (124/186) of persons who received transnational services belonged to the subgroup that was detained before removal.

**Table 4:** Estimated number of persons with tuberculosis referred for transnational care–continuity services to prevent treatment interruption due to voluntary or involuntary departure from the United States, 2008–2012

Variable	Total no. estimated cases among study population*	No. cases managed by CureTB†	No. cases managed by Health Network
Year			
2008	2,659	90	106
2009	2,430	111	95
2010	2,853	108	109
2011	3,099	111	134
2012	3,094	90	155
Annual average (% referred)	2,827 (8)	102	120
Total incident cases from detained and removed subgroup (% referred)	928 (67)	180	442
Annual average for subgroup	186	36	88

*Study population was defined as nonimmigrants, nonrefugees who were born outside of the United States, had active tuberculosis while in the United States, and then left the United States before treatment completion was possible. †Numbers for 2008 and 2009 were extrapolated by using previously reported data from 2010–2012 (*27**,**28*).

## Discussion

We estimated that, during 2008–2012, a substantial number of TB-infected persons were at risk for drug resistance because of treatment interruption due to departure from the United States. During that time, 14,134 cases of incident TB occurred among visitors to the United States, representing a yearly average of 2,827 cases (2,586 and 241, respectively, among persons with and without authorization). Approximately 10% of these persons received transnational care–continuity services (from Health Network or CureTB). Thus, ≈90% of infected persons departed the country without such services, a finding that highlights a neglected public health area and the feasibility of scaling up intervention.

Pathogens that cause TB are transmitted via breathing, and the disease has a high death rate if untreated (*29*), thereby incurring severe negative externalities for the public’s health and economic wellbeing (*30*). A single untreated case can lead to hundreds of new infections (*31*,*32*). If treatment is interrupted, the situation is worsened because of the risk for poorer outcomes (*29*). Our findings contribute to TB control efforts by elucidating characteristics of an understudied population at risk for acquiring and spreading drug-resistant TB (*6*) and by highlighting opportunities to prevent this serious threat to the public’s health and the corresponding fiscal consequences. Moreover, our findings contribute to previously identified needs for improving screening practices for migrants (*33*) and for understanding how best to target TB prevention and control efforts (*7*). Our findings build on those of Liu et al. (*11*), particularly the finding that temporary residents contribute appreciably to illness in the United States caused by TB; the reported number of TB cases in 2012 was 9,945, of which 6,274 were among foreign-born persons (*8*). Our estimate of 2,827 yearly cases among visitors to the United States does not entirely overlap with the number from that report because we counted persons with <90 days of treatment (*34*) and we captured undiagnosed cases.

Little is known about TB cases among subpopulations living in the United States without authorization. The attribution of only 8.5% of cases to this subgroup contradicts widespread opinion that TB in the United States is primarily due to illegal immigration. Moreover, 8.5% is consistent with the finding in a multinational study (*35*). A county-level study found 25% of TB cases in the unauthorized population (*36*), but it is difficult to generalize from a single county’s data. A related and somewhat encouraging finding was that 67% (124/186) of persons receiving transnational services were among the most vulnerable subgroup (those detained before removal). Ideally, no one would be forcibly relocated until after treatment completion (*35*), but assuring all who are removed receive transnational services is another way to avoid treatment interruption and development of drug-resistant TB. Our findings suggest that scaling up transnational care-continuity services is feasible and desirable, given the likely return on investment (*9*,*30*). Furthermore, removal of unauthorized visitors from the United States has been increasing over the past decade (*25*), suggesting incident TB cases among this subgroup will remain at estimated levels or decrease in future years. The Obama administration’s executive action in November 2014 to provide immigration relief to specific persons without authorization to enter the United States may slightly reduce this estimate because it temporarily halts deportation.

The authorized subgroups differ from each other, just as the unauthorized subgroups differ in risk and migratory profiles. Therefore, here we consider program and policy implications separately by subgroup. First, we concur with the suggestion by Liu et al. (*11*) to prescreen only subgroups that have the highest case rates per 100,000 admissions and are in the United States long enough to make postarrival medical follow-up feasible and worthwhile. This policy would affect students, exchange visitors, and temporary workers from countries with high TB incidences and expand the successful prescreening–plus–follow-up policy for immigrants and refugees (*37*). If persons in these subgroups do not stay in the United States long enough to complete treatment, they should be referred for transnational care–continuity services. Any compulsory screening program must be accompanied by regard for civil liberties and medical ethical principles (*6*). In addition, some persons with TB who leave the country complete treatment without the aid of transnational services; however, case management increases the likelihood of completion, and US-based providers would have more data should a patient return, a probable occurrence for many (*9*).

Second, diplomats and other representatives from high-incidence countries also had a relatively high TB case rate, but the number of admissions was not sufficient to make prescreening a high-yield activity. Political calculus also weighs heavily for this group of visitors, and diplomats tend to have preexisting mechanisms for health emergencies. Therefore, further intervention is impractical or unnecessary.

Third, when a large volume of admissions to the United States and relatively low TB case rates are combined, referral to transnational care–continuity services after TB diagnosis is more rational than prescreening. Subgroups falling into this category are tourist and business travelers; persons from Canada and Mexico entering without an I-94 card; and any authorized visitor from a country with medium or low TB incidence, except for diplomats.

Last, subgroups without authorization to enter the United States have little interaction with formal systems that would help to identify and treat their TB infections in a timely manner. This situation is especially true in the wake of the Affordable Care Act of 2010, which prohibited such persons from purchasing private health insurance (*38*). The best option in this circumstance is to refer unauthorized visitors for transnational services immediately after they are diagnosed with TB. Persons who are detained by immigration officials are typically screened for TB (*17*); this practice should continue, as it increases the chances of referral for transnational care–continuity services.

Our study had limitations. First, there were time lags in DHS data (*16*), thus, where available, we used postadjustment numbers for removal totals. Also, in 2010, DHS started counting all visa admissions separately rather than counting multiple entries for 1 person as 1 admission. An increase resulted, particularly among admissions from Canada and Mexico (*12*), suggesting that estimates from 2009 and earlier were biased toward undercounting. This change also represents the second biggest factor in the difference between our estimate and those from previous studies (*11*). Nevertheless, given the affected subgroups, the policy implications do not change.

Second, there was uncertainty around the time at risk for TB. However, our sensitivity analyses varied this input in both directions for the unauthorized subpopulations, and the findings remained robust. For the authorized subpopulations with an I-94 card, substituting median LOS for mean LOS dramatically reduced time at risk. The overall estimate was nearly halved, but the order of magnitude was the same, as do intervention recommendations, with the exception that prescreening for temporary workers from high-incidence countries might no longer be a high-yield intervention. Furthermore, the available LOS data are highly suggestive of smooth skews rather than random outliers with problematic influence (*13*); thus, the best way to statistically account for those days at risk is by using mean LOS.

Third, a conservative bias was introduced by global TB underreporting (*39*), which affected the estimated number of cases and corresponding CIs. A countervailing bias was introduced by not adjusting for visitor socioeconomic status or age upon US entry because of insufficient data. Moreover, data from our sources were consistent with those in similar studies (*11*). Additional bias toward overcounting occurred due to lack of data on visitors who adjusted status to permanent residency, for whom TB screening is required. Because most of those who adjust status come from the group for whom we recommended preentry screening and postentry follow up, our recommendation remains unchanged and would aid visitors who adjust their status, because they will have completed their TB screening early.

The 2,827 annual cases would include some drug-resistant TB cases, depending on the strain contracted. Drug-resistant and MDR TB lend urgency to achieving treatment completion; however, without additional mechanisms besides the international referral form, US clinicians and health departments rarely know outcomes for patients exiting the country. In contrast, CureTB and Health Network have documented completion rates, approaching the WHO target of 85% (*20*,*21*,*24*). Therefore, our recommendation to refer these patients for transnational services is justified in order to reduce the number and spread of these deadly and costly conditions.

In summary, TB in mobile persons in the United States is not well understood and represents a particular challenge to global TB control (*6*), as well as a key opportunity to reduce development and spread of drug-resistance. Our findings provide new epidemiologic evidence that will inform an effective TB control strategy (*6*). Because many mobile persons with TB may return to the United States (*9*) and the global prevalence of MDR TB is increasing (*4*,*24*), scaling up transnational care–continuity services would benefit the US directly and bolster international TB control efforts (*40*). Use of such services of would reduce suffering, save lives, build goodwill with receiving countries, improve global TB surveillance data, and bolster economic productivity. Access to healthcare varies among subgroups of mobile, TB-infected persons; however, programs like CureTB and Health Network are able to serve all subgroups. The most complete policy response may be to make these services available to public and private clinicians alike.

Technical Appendix Further discussion and data on methodology, sensitivity analyses, and study limitations.

## References

[1] Marks SM, Flood J, Seaworth B, Hirsch-Moverman Y, Armstrong L, Mase S, Treatment practices, outcomes, and costs of multidrug-resistant and extensively drug-resistant tuberculosis, United States, 2005–2007. Emerg Infect Dis. 2014;20:812–21. 24751166 10.3201/eid2005.131037 PMC4012799

[2] Shin SS, Furin JJ, Alcantara F, Bayona J, Sanchez E, Mitnick CD. Long-term follow-up for multidrug-resistant tuberculosis. Emerg Infect Dis. 2006;12:687–8. 10.3201/eid1204.041256 16704823 PMC3294679

[3] Castro KG, LoBue P. Bridging implementation, knowledge, and ambition gaps to eliminate tuberculosis in the United States and globally. Emerg Infect Dis. 2011;17:337–42. 10.3201/eid1703.110031 21392421 PMC3166034

[4] Zumla A, Abubakar I, Raviglione M, Hoelscher M, Ditiu L, Mchugh TD, Drug-resistant tuberculosis—current dilemmas, unanswered questions, challenges, and priority needs. J Infect Dis. 2012;205(Suppl 2):S228–40. 10.1093/infdis/jir858 22476720

[5] Abarca Tomás B, Pell C, Bueno Cavanillas A, Guillén Solvas J, Pool R, Roura M. Tuberculosis in migrant populations. A systematic review of the qualitative literature. PLoS ONE. 2013;8:e82440. 10.1371/journal.pone.0082440 24349284 PMC3857814

[6] Dhavan P, Mosca D. Tuberculosis and migration: a post-2015 call for action. Migration Policy Practice Journal. 2014;IV:17–22 [cited 2014 Nov 10]. http://www.iom.int/files/live/sites/iom/files/What-We-Do/docs/MPP15_TB.pdf

[7] Pareek M, Baussano I, Abubakar I, Dye C, Lalvani A. Evaluation of immigrant tuberculosis screening in industrialized countries. Emerg Infect Dis. 2012;18:1422–9. 10.3201/eid1809.120128 22931959 PMC3437731

[8] Centers for Disease Control and Prevention. Reported tuberculosis in the United States, 2012. 2013 [cited 2014 Nov 17]. http://www.cdc.gov/tb/statistics/reports/2012/pdf/report2012.pdf

[9] Centers for Disease Control and Prevention. Post-detention completion of tuberculosis treatment for persons deported or released from the custody of the Immigration and Naturalization Service—United States, 2003. MMWR Morb Mortal Wkly Rep. 2003;52:438–41 . 12807085

[10] World Health Organization. Tuberculosis (TB). 2014 [cited 2014 Jan 1]. http://www.who.int/tb/country/data/download/en/index.html

[11] Liu Y, Painter JA, Posey DL, Cain KP, Weinberg MS, Maloney SA, Estimating the impact of newly arrived foreign-born persons on tuberculosis in the United States. PLoS ONE. 2012;7:e32158. 10.1371/journal.pone.0032158 22384165 PMC3287989

[12] US Department of Homeland Security. 2012 yearbook of immigration statistics. 2013 Jul [cited 2014 Nov 17]. http://www.dhs.gov/publication/yearbook-2012

[13] Grieco EM. Length of visit of nonimmigrants departing the United States in 2003. 2005 [cited 2014 Oct 5]. http://www.dhs.gov/xlibrary/assets/statistics/publications/LengthVstNonim2003.pdf

[14] Grieco EM. Estimates of the nonimmigrant population in the United States: 2004. 2006 [cited 2014 Oct 5]. http://www.dhs.gov/xlibrary/assets/statistics/publications/NIM_2004.pdf

[15] United States Department of State. U.S. visas: Nonimmigrant visa statistics. 2014 [cited 2014 Aug 21]. https://travel.state.gov/content/dam/visas/Statistics/Non-Immigrant-Statistics/NIVDetailTables/FY14NIVDetailTable.xls</eref>

[16] United States Department of Homeland Security. Immigration statistics publications. Annual reports: immigration enforcement actions, 2008–2012 [cited 2014 Oct 5]. http://www.dhs.gov/immigration-statistics-publications

[17] Schneider DL, Lobato MN. Tuberculosis control among people in U.S. Immigration and Customs Enforcement custody. Am J Prev Med. 2007;33:9–14. 10.1016/j.amepre.2007.02.044 17572305

[18] Passel JS, Cohn DV, Gonzalez-Barrera A. Net migration from Mexico falls to zero—and perhaps less. 2012 Apr 23 [cited 2014 Nov 17]. http://www.pewhispanic.org/files/2012/04/Mexican-migrants-report_final.pdf

[19] BBVA Economic Studies Service (Mexico). Migration situation in Mexico [in Spanish]. 2012 Jul [cited 2014 Sept 4]. https://www.bbvaresearch.com/wp-content/uploads/migrados/1207_SitMigracionMexico_Jul12_tcm346-344007.pdf

[20] Zuroweste E. TBNet stats 2005–2012. Presented at: Annual Meeting of the National Tuberculosis Controllers Association; 2014 Jun 11–14; Atlanta, Georgia, USA.

[21] Vera-Garcia C. Managing cases across borders: US, Mexico and Central America. Presented at: 18th Annual Conference, International Union against Tuberculosis and Lung Disease–North America Region; 2014 Feb 26–Mar 1; Boston, Massachusetts, USA.

[22] Cain KP, Haley CA, Armstrong LR, Garman KN, Wells CD, Iademarco MF, Tuberculosis among foreign-born persons in the United States: achieving tuberculosis elimination. Am J Respir Crit Care Med. 2007;175:75–9. 10.1164/rccm.200608-1178OC 17038659

[23] Walter ND, Painter J, Parker M, Lowenthal P, Flood J, Fu Y, Persistent latent tuberculosis reactivation risk in United States immigrants. Am J Respir Crit Care Med. 2014;189:88–95 . 24308495 10.1164/rccm.201308-1480OC PMC3919127

[24] World Health Organization. Global tuberculosis report 2013. 2013 Nov [cited 2014 Nov 17]. http://apps.who.int/iris/bitstream/10665/137094/1/9789241564809_eng.pdf?ua=1

[25] Bojorquez I, Aguilera R, Ramírez J, Cerecero D, Mejía S. Common mental disorders at the time of deportation: a survey at the Mexico–United States border. J Immigr Minor Health. 2015;17:1732–8. 10.1007/s10903-014-0083-y 25118675

[26] Rosales C, Ortega MI, De Zapien JG, Paniagua ADC, Zapien A, Ingram M, The US/Mexico border: A binational approach to framing challenges and constructing solutions for improving farmworkers’ lives. Int J Environ Res Public Health. 2012;9:2159–74. 10.3390/ijerph9062159 22829796 PMC3397370

[27] Combellick J, Zuroweste E, Gany FM. TBNet: the impact of an innovative public–private intervention on tuberculosis control among an internationally mobile population. J Immigr Refug Stud. 2011;9:229–41. 10.1080/15562948.2011.592805

[28] Moser K. CureTB: CureTB US/Mexico tuberculosis referral and information program. Presented at: 16th Annual Conference, International Union against Tuberculosis and Lung Disease–North America Region; 2012 Feb 22–25; San Antonio, Texas, USA.

[29] Zumla A, Raviglione M, Hafner R, Fordham von Reyn C. Tuberculosis. N Engl J Med. 2013;368:745–55. 10.1056/NEJMra1200894 23425167

[30] Jack W. The public economics of tuberculosis control. Health Policy. 2001;57:79–96 and. 10.1016/S0168-8510(01)00140-3 11395176

[31] Walker TM, Ip CLC, Harrell R, Evans J, Kapatai G, Dedicoat M, Whole-genome sequencing to delineate *Mycobacterium tuberculosis* outbreaks: a retrospective observational study. Lancet Infect Dis. 2013;13:137–46. 10.1016/S1473-3099(12)70277-3 23158499 PMC3556524

[32] Garcia D, Wares F, Zuroweste E, Guerin P. Tuberculosis and migration. In: Schaaf HS, Zumla AI, editors. Tuberculosis: a comprehensive clinical reference. Philadelphia: Saunders Elsevier; 2009. p. 892–900.

[33] Alvarez GG, Gushulak B, Rumman KA, Altpeter E, Chemtob D, Douglas P, A comparative examination of tuberculosis immigration medical screening programs from selected countries with high immigration and low tuberculosis incidence rates. BMC Infect Dis. 2011;11:3. 10.1186/1471-2334-11-3 21205318 PMC3022715

[34] Centers for Disease Control and Prevention. CDC tuberculosis surveillance data training. Report of verified case of tuberculosis. Instruction manual. 2010 [cited 2014 Oct. 5]. http://www.cdc.gov/TB/programs/rvct/InstructionManual.pdf

[35] Heldal E, Kuyvenhoven JV, Wares F, Migliori GB, Ditiu L, Fernandez de la Hoz K, Diagnosis and treatment of tuberculosis in undocumented migrants in low- or intermediate-incidence countries. Int J Tuberc Lung Dis. 2008;12:878–88 . 18647446

[36] Weis SE, Moonan PK, Pogoda JM, Turk LE, King B, Freeman-Thompson S, Tuberculosis in the foreign-born population of Tarrant County, Texas by immigration status. Am J Respir Crit Care Med. 2001;164:953–7. 10.1164/ajrccm.164.6.2102132 11587977

[37] Liu Y, Weinberg MS, Ortega LS, Painter JA, Maloney SA. Overseas screening for tuberculosis in U.S.-bound immigrants and refugees. N Engl J Med. 2009;360:2406–15. 10.1056/NEJMoa0809497 19494216

[38] Vargas Bustamante A, Fang H, Garza J, Carter-Pokras O, Wallace S, Rizzo J, Variations in healthcare access and utilization among Mexican immigrants: The role of documentation status. J Immigr Minor Health. 2012;14:146–55. 10.1007/s10903-010-9406-9 20972853 PMC3256312

[39] World Health Organization. Assessing tuberculosis under-reporting through inventory studies. 2012 [cited 2014 Nov 17]. http://www.who.int/iris/bitstream/10665/78073/1/9789241504942_eng.pdf?ua=1

[40] Schwartzman K, Oxlade O, Barr RG, Grimard F, Acosta I, Baez J, Domestic returns from investment in the control of tuberculosis in other countries. N Engl J Med. 2005;353:1008–20. 10.1056/NEJMsa043194 16148286

