# Supplementary material for: Use of Transnational Services to Prevent Treatment Interruption in Tuberculosis-Infected Persons Who Leave the United States
Source: Emerg Infect Dis. 2016 Mar;22(3):417–25. doi: 10.3201/eid2203.141971 (PMC4766910; doi:10.3201/eid2203.141971)
Supplement: Technical Appendix — Further discussion and data on methodology, sensitivity analyses, and study limitations. [file 14-1971-Techapp-s1.pdf]

Article DOI: http://dx.doi.org/10.3201/eid2203.141971

Use of Transnational Services to Prevent Treatment Interruption in Tuberculosis-Infected Persons Who Leave the United States

Technical Appendix

Median Length of Stay versus Mean Length of Stay

The primary reason for our estimates of incident TB cases being greater than similar studies (*1*) is our methodological choice to use mean length of stay (LOS) rather than the median LOS. The challenge was to balance legitimate concern about the influence of very long stays with the desire to capture all time-at-risk for as accurate an estimate of TB cases as possible. The main limitation to using median LOS is that it does not use the data at the end of a skewed distribution. The main limitation to using mean LOS is that outliers can create noise in one's calculations. We compared the means versus medians (Appendix Table 1), as well as the frequency distributions (i.e., stays for <six months, 6–12 months, and >one year) for all the visa categories where data was available (*2*). There is some variation in the ratios between the means and medians, but the evidence is most supportive of smooth distributions with a skew rather than outliers with undo influence. For example, the B-2 visa class (i.e., temporary visitors for pleasure) was weighted using 5.6 million records. The mean LOS was 48 days and the median was 13. While that represents a dramatic ratio of nearly 4:1, there were 7.2% that stayed between 6 months to a year, or significantly longer than 13 days. Moreover, nearly 1%, or 45,000 persons, stayed for over a year, representing ≈45,000 person-years that would not be captured by the use of the median LOS.

Finally, the calculations involve aggregates of 202,766 to 12,199,633 records per subgroup. This large volume is protective against noise introduced by outliers. Therefore, we maintain that the best statistical approach to our person-year calculations was to use mean LOS, as this allowed us to capture the impact of the notable proportions of people staying substantially

longer than their peers in each visa category. Nevertheless, we are very cognizant that our estimates seem high, particularly those familiar with the U.S. TB surveillance system. Thus, we recalculated every estimate for which a weighted medial LOS was available (see next section).

We also investigated another aspect as to why our estimates are higher than Liu et al., specifically the impact of the Department of Homeland Security's 2010 change in counting admissions methodology. In short, more admissions were counted, rather than being obscured by the fact that some visas allow multiple entries, and therefore, multiple times at risk for TB activation. For example, the "tourists and business travelers" (with a visa) subgroups had a weighted median LOS of 6.7 and 11.2 days, respectively in our calculations, and 7 days in those of Liu and colleagues. This led to 203 (199 TB and 4 MDR TB) annual cases from 552,984 person-years at risk in their estimates, while we calculated 379 for 1,006,560 person-years at risk. In contrast, for the "diplomats and other representatives" subgroup – predominantly unaffected by the change in counting methodology – we found 13 TB cases and Liu et al. estimated 11.

Sensitivity Analyses for Subgroups with Authorization

Our first sensitivity analyses for subgroups residing in the U.S. with authorization involved replacing the weighted mean LOS with a weighted median LOS where available (see Appendix Table 1 for a complete list) and holding all other methodology constant. The result was a total of 7,722 TB cases and an annual average of 1,544 cases (95% CI: 1,249–1,840). This means that TB cases among those without authorization would make up 15.6% (241/1,544) and those that received transnational care continuity and management services represented 14% (222/1,544) of total estimated cases (Appendix Tables 3–6).

We conducted another set of sensitivity analyses using visa count data from the U.S. Department of State (DOS). This provided an alternative to the 95% CI by calculating the range within which the actual number of incident cases should fall by adapting our primary person-years equation (equation 1, Table 1). For the lower bound, we assumed an average of only one admission per visa (despite multiple-entry visas) and replaced admissions with visa counts, stratified by country and year. For the upper bound we assumed each visa-holder had 12 months of risk, the highest possible value. When using the mean LOS for the visas, we found a

cumulative range of 6,761–23,186 cases, for an annual range of 1,352–4,637. After substituting median LOS where available, the annual range shifted to 649–4,637. The upper bound did not shift because in both cases, the maximum time-at-risk was 12 months. Both of these ranges are confirmatory of our findings.

Sensitivity Analyses for Subgroups without Authorization

Passel and Cohen provided data on the amount of time Mexican nationals spent in the U.S. before being removed by U.S. officials (*3*). We took the midpoints and percentages of their three categories (i.e., greater than a year, between 1 year and 1 week, and less than a week) and calculated a weighted mean length of stay of 122 days. We then assumed that people from other nations would have a greater proportion staying in the U.S. longer because they invest more to arrive in the U.S. and immigration enforcement trends strongly discourage returning to one's home country for any reason other than by force (*3*–*5*). Thus, we assumed 50% were here more than a year and 50% were here less than a week, resulting in an estimated weighted mean stay of 186 days. We then combined these two means into a single value and multiplied the proportion of 2010 removals involving Mexicans versus all other countries (*6*). This weighted mean, i.e., 140 days, served as the time-at-risk for all persons being removed by U.S. officials. These three estimates of time-at-risk were then substituted in for the base-case assumption of 6 months (i.e., 183 days) at risk among all unauthorized visitors. We also estimated TB incident cases at 9 months of risk as a further sensitivity analysis, because it is possible that someone suffered a delay in diagnosis or a treatment interruption, began again, and then left the U.S. It is also possible that if the patient's TB is complex in any way, the treatment course will be closer to 9 months, increasing the time-at-risk for treatment interruption.

The American Civil Liberties Union of Massachusetts (ACLUM) and the Transactional Records Access Clearinghouse (TRAC) Immigration tools provided key data on those detained by U.S. immigration officials (*4,7,8*). We expected to use their estimates of average time detained (for various portions of those detained) to adjust the time-at-risk parameter for our sensitivity analyses involving those present without authorization. Ultimately, there was not enough data to provide a viable substitute for additional sensitivity analyses.

There was limited evidence that the removal rate of those with TB who were first detained by immigration officials was closer to 69% (*7*) rather than our base case of 80%. Applying the 69% rate, we estimated 165 fewer cases among that subgroup (i.e., 763 over the same 5-year period). However, the data was too limited, i.e., it applied to only 1 month's worth of data for this subgroup, to report this removal rate rather than the one used from Schneider & Lobato (*9*).

The TB case rate among those detained by U.S. immigration officials held some uncertainty as well. Schneider and Lobato reported a "TB case rate" of 83 per 100,000 person-years in 2004 and 122/100,000 in 2005 (*9*), giving a weighted mean of 108/100,000. However, in the article text, it seemed that the case rate might have been a prevalence rate rather than an incidence rate, which is what we needed for our estimate of incident TB cases. We estimated a new case rate based on the equation of [prevalence = incidence x duration]. The duration of untreated TB is typically cited at 2 years (*10*), although there is some evidence of duration of 3 years in non-HIV-infected persons (*11*). The sensitivity analysis for this subgroup involved a TB case rate range of 36–64 per 100,000 person-years, yielding an estimated 339–496 incident TB cases, which was unrealistically low based on actual cases referred for transnational care-continuity services (i.e., an estimated 622 cases). Therefore, we proceeded with the weighted mean of 108/100,000.

Calculating Confidence Intervals

There were multiple steps to calculate our 95% confidence intervals (CI), which we describe here in greater detail. First, we calculated the initial estimate of incident TB cases among the population present in the U.S. with authorization for each country, year, and visa grouping. We then aggregated the TB cases into subtotals according to low-, medium-, and high-burden levels within each visa grouping (plus the Canadian/Mexican without an I-94 category) for each of the 5 years, 2008–2012. We then assumed a Poisson distribution to each subtotal, which equated those incidence numbers with the mean of the distribution. They also equaled the variance since, for a Poisson distribution, the mean is equal to the variance. Taking the square root of the variance gave the standard deviation. With the standard deviation, we calculated the

CIs for each subtotal by adding and subtracting to the mean the product of 1.96 times the standard deviation.

The next step was to add the distributions, since adding two Poisson distributions creates another Poisson distribution. Practically speaking, this meant we added the incident TB cases (i.e., the means), the lower bounds of TB cases (i.e., the means minus 1.96 times standard deviations), and the upper bounds of TB cases (i.e., the means plus 1.96 times standard deviations) for an estimated number of incident TB cases with 95% CIs for each year. By adding all the lower bounds together and all the upper bounds together, we obtained a conservative (i.e., wide) CI, which we thought appropriate given the large number of assumptions in our study.

At this point, we did not add the 5 year totals together since another underlying assumption of the Poisson distribution is that the events are independent, and the WHO's TB incidence rates for each country are related year-to-year. Therefore, we opted to apply the 2010 proportion of the CI width of the mean to the final 5-year annual case estimate for the entire study population (Appendix Table 2). This step again required adding the lower bound of the authorized subpopulation to the lower bound of the unauthorized subpopulation and adding the two upper bounds. The result was a wider CI, which we felt would better serve public health officials as they consider the response that might be needed for this new surveillance population we investigated. It is worth noting that the lower and upper bound estimates for the unauthorized population were not calculated using a Poisson distribution due to the fragmented nature of the available data. Rather, we obtained a realistic range through the sensitivity analyses described above, specifically, by varying the time-at-risk.

U.S.–Mexico Border Flow

In general our study subgroups represent a comprehensive and mutually exclusive set of categories with regard to foreign-born individuals spending time in the U.S. There was one additional group that may have had overlap with one or more of our subgroups, specifically those that cross the U.S.-Mexico border multiple times a year. This category should generally only affect those without authorization, since the visa holders and the Mexican visitors without an I-94 card likely accounted for all of those that "flow" across the border with authorization. That being the case, we expected the number to be very small given the increased militarization of the

border since the Illegal Immigration Reform and Immigrant Responsibility Act of 1996. Due to the expected small numbers and the paucity of data, we did not attempt to calculate TB cases for that subgroup separately. We also did not include the possible contributions of various bi-national programs along the U.S.-Mexico border, e.g., Los Dos Laredos and Grupo Sin Fronteras (*12*). This is because they predominately deal with cases that do not leave the U.S. so much as cross back and forth or cases that remain in one country but had contacts located in the other. Furthermore, the estimated 30 (range, 0–60) cases that would fit into the current study's definition do no change the 8% proportion receiving transnational care-continuity services.

Accounting for Differences in Socio-economic Status and Age

For the subgroups in the U.S. with authorization, there was the concern that applying the country TB incidence rate to all nonimmigrant visitors would overestimate the TB case rates among the visitors with higher socio-economic status (SES) (*13*). For example, Buskin et al. found that having one indicator of low SES led to 3.0 times the odds of having TB (*14*). While it is true that TB has long been associated with poverty, the pathways are more complicated than simply having higher income or more education. Davidow and colleagues discussed how many TB cases in New Jersey were found among those who were educated, employed, and living in an affluent locations (*15*). Nahid and colleagues compared TB risk between Blacks and Whites and found that SES did not explain the racial disparity in TB (*16*), which contradicts earlier findings that SES factors accounted for more than half of the increased risk for TB among minority groups in the U.S (*17*). Holtgrave & Crosby found social capital to be the strongest predictor variable in analyses that included poverty and inequality (*18*). Lonnroth and colleagues suggested that the causal pathways have more to do with links between SES and proximate risk factors, i.e., those factors that increase exposure to infected droplets or decrease the host's defense against infection (*13*). Urbanization is a particularly potent risk factor for TB, which would impact people from most SES levels (*13*). Mitnick et al. found that TB risk based on origination from a high-income country was modified by conditions related to SES, particularly crowded living quarters (*19*); similarly, risk based on origination from a low-income country was mitigated by indicators of social support and access (*19*). This suggests that our chosen TB rates are both consistent with the literature (*1*), and that the biases due to SES differences may balance

out since those with low SES from low-burden countries would cause some undercounting and those with high SES from high-burden countries would cause some over-counting.

A similar situation arose from that fact that we did not have enough data to account for age upon U.S. entry. Cain et al. found different incident TB case rates among distinct age cohorts, with increasing rates as age grew (*20*). If the admissions data had been aggregated by age as well, then the estimated TB case rates her would likely be slightly lower. Nevertheless, we do not think this had a substantial impact on the findings because the majority of our study population was made up of business visitors, tourists, students, and temporary workers, thus the overwhelming majority fell into the 18–45 year-old range. This age range showed the most constant TB case rates in the Cain et al. study (*20*). Furthermore, our study implications and recommendations are robust whether the actual number of TB cases is somewhat greater or less than our estimate.

Other notes

The majority of TB morbidity in the U.S. is imported because TB is still endemic in most of the world (*21*), while the U.S. has pursued TB control aggressively. However, since the majority of foreign visitors and residents to the U.S. also enter legally, the majority of imported TB follows that migratory pattern.

For any of the subgroups studied here, enrolling the patients in programs like Health Network or CureTB also has implications for domestic and global TB surveillance efforts. For example, Health Network updates the referring clinician after a final TB treatment outcome has been confirmed with the patient and the final treating clinician. An expansion of cases being managed after leaving a nation should make more data available regarding the magnitude and dynamics of TB on the move. Domestically, Health Network's services are also available for any TB case mobile within the U.S., and could serve as the necessary link between the clinician first identifying each case and public health officials (*22*).

The reported number of TB cases in 2012 was 9,945, of which 6,274 were foreign-born (*23*). Our yearly estimate of 2,827 would not entirely overlap with CDC's reported number because of the policy of not counting cases with fewer than 90 days of treatment (*24*), and our calculations capture undiagnosed cases. It is worth noting that a 2010 revision to the CDC report

form may improve surveillance data for those who leave the U.S. before completing 90 days of treatment once all jurisdictions utilize the revised portions fully (*24*).

References

1. Liu Y, Painter JA, Posey DL, Cain KP, Weinberg MS, Maloney SA, et al. Estimating the impact of newly arrived foreign-born persons on tuberculosis in the United States. PLoS ONE. 2012;7:e32158. PubMed http://dx.doi.org/10.1371/journal.pone.0032158

2. Grieco EM. Length of visit of nonimmigrants departing the United States in 2003. Washington (DC): US Department of Homeland Security, Office of Immigration Statistics; 2005.

3. Passel JS, Cohn DV. Net migration from Mexico falls to zero—and perhaps less. Washington (DC): Pew Hispanic Center; 2012 April 23 [cited 2014 Nov 17]. http://www.pewhispanic.org/files/2012/04/Mexican-migrants-report_final.pdf

4. American Civil Liberties Union of Massachusetts. Detention and deportation in the age of ICE: Immigrants and human rights in Massachusetts. Boston (MA): ACLUM; 2008 December.

5. Rosales C, Ortega MI, De Zapien JG, Paniagua ADC, Zapien A, Ingram M, et al. The US/Mexico Border: A binational approach to framing challenges and constructing solutions for improving farmworkers' lives. Int J Environ Res Public Health. 2012;9:2159–74. PubMed http://dx.doi.org/10.3390/ijerph9062159

6. United States Department of Homeland Security. Immigration enforcement actions: 2010. Annual Report. Washington (DC): USDHS, Office of Immigration Statistics; 2011 June.

7. Transactional Records Access Clearinghouse. Legal noncitizens receive longest ICE detention. TRAC Inc.; 2013.

8. TRAC Immigration Tools. TRAC Inc.; 2014 [cited 2014 Aug 21]. http://trac.syr.edu/phptools/reports/reports.php?layer=immigration&report_type=tool

9. Schneider DL, Lobato MN. Tuberculosis control among people in U.S. immigration and customs enforcement custody. Am J Prev Med. 2007;33:9–14. PubMed http://dx.doi.org/10.1016/j.amepre.2007.02.044

10. Ait-Khaled N, Enarson DA. Tuberculosis: A manual for medical students. Geneva: WHO and the International Union against TB and Lung Disease; 2003.

11. Tiemersma EW, van der Werf MJ, Borgdorff MW, Williams BG, Nagelkerke NJD. Natural history of tuberculosis: Duration and fatality of untreated pulmonary tuberculosis in HIV negative patients: A systematic review. PLoS ONE. 2011;6:e17601. PubMed http://dx.doi.org/10.1371/journal.pone.0017601

12. Centers for Disease Control and Prevention. Preventing and controlling tuberculosis along the U.S.–Mexico border. MMWR Recomm Rep. 2001;50:1–27. PubMed

13. Lönnroth K, Jaramillo E, Williams BG, Dye C, Raviglione M. Drivers of tuberculosis epidemics: The role of risk factors and social determinants. Soc Sci Med. 2009;68:2240–6. PubMed http://dx.doi.org/10.1016/j.socscimed.2009.03.041

14. Buskin SE, Gale JL, Weiss NS, Nolan CM. Tuberculosis risk factors in adults in King County, Washington, 1998 through 1990. Am J Public Health. 1994;84:1750–6. PubMed http://dx.doi.org/10.2105/AJPH.84.11.1750

15. Davidow AL, Mangura BT, Napolitano EC, Reichman LB. Rethinking the socioeconomics and geography of tuberculosis among foreign-born residents of New Jersey, 1994–1999. Am J Public Health. 2003;93:1007–12. PubMed http://dx.doi.org/10.2105/AJPH.93.6.1007

16. Nahid P, Horne DJ, Jarlsberg LG, Reiner AP, Osmond D, Hopewell PC, et al. Racial differences in tuberculosis infection in United States communities: The coronary artery risk development in young adults study. Clin Infect Dis. 2011;53:291–4. PubMed http://dx.doi.org/10.1093/cid/cir378

17. Cantwell MF, McKenna MT, McCray E, Onorato IM. Tuberculosis and race/ethnicity in the United States: Impact of socioeconomic status. Am J Respir Crit Care Med. 1998;157:1016–20. PubMed http://dx.doi.org/10.1164/ajrccm.157.4.9704036

18. Holtgrave DR, Crosby RA. Social determinants of tuberculosis case rates in the United States. Am J Prev Med. 2004;26:159–62. PubMed http://dx.doi.org/10.1016/j.amepre.2003.10.014

19. Mitnick C, Furin J, Henry C, Ross J. Tuberculosis among the foreign born in Massachusetts, 1982–1994: a reflection of social and economic disadvantage. Int J Tuberc Lung Dis. 1998;2:S32–40. PubMed

20. Cain KP, Benoit SR, Winston CA, Mac Kenzie WR. Tuberculosis among foreign-born persons in the United States. JAMA. 2008;300:405–12. PubMed http://dx.doi.org/10.1001/jama.300.4.405

21. World Health Organization. Global tuberculosis report 2013 [cited 2014 Nov 17]. http://apps.who.int/iris/bitstream/10665/137094/1/9789241564809_eng.pdf?ua=1

22. White MC, Tulsky JP, Menendez E, Goldenson J, Kawamura LM. Incidence of TB in inmates with latent TB infection: 5-year follow-up. Am J Prev Med. 2005;29:295–301. PubMed http://dx.doi.org/10.1016/j.amepre.2005.06.014

23. Centers for Disease Control and Prevention. Reported tuberculosis in the United States, 2012. 2013 [cited 2014 Nov 17]. http://www.cdc.gov/tb/statistics/reports/2012/pdf/report2012.pdf

24. Centers for Disease Control and Prevention. CDC tuberculosis surveillance data training - Report of verified case of tuberculosis instruction manual. 2010 [cited 2014 October 5]. http://www.cdc.gov/TB/programs/rvct/InstructionManual.pdf

25. Vera-Garcia C. Managing cases across borders: US, Mexico and Central America. Presented at: 18th Annual Conference, International Union against Tuberculosis and Lung Disease–North America Region; 2014 Feb 26–Mar 1; Boston, Massachusetts, USA.

26. Moser K. CureTB: CureTB US/Mexico tuberculosis referral and information program. Presented at: 16th Annual Conference, International Union against Tuberculosis and Lung Disease–North America Region; 2012 Feb 22–25; San Antonio, Texas, USA.

Technical Appendix Table 1. Weighted Median and Mean Lengths of Stay and Ratios for Nonimmigrant Visa Types by Visa Grouping*

Visa type	No.† with visa type	Median LOS for visa type (days)	Weighted median LOS (days)	Mean LOS for visa type (days)	Weighted mean LOS (days)	Ratio, wt. mean to wt. median
Visa waiver						
GB	1,201	4	0.0	4	0.0	1.0
GMB	n/a	n/a	n/a	n/a	n/a	n/a
GT	87,697	3	0.0	4	0.0	1.3
GMT	n/a	n/a	n/a	n/a	n/a	n/a
WB	1,827,070	5	0.7	8	1.2	1.6
WT	10,283,665	7	5.9	11	9.3	1.6
Subgroup total	12,199,633		6.7		10.5	
Business and tourist travelers						
B-1	1,889,980	6	1.5	15	3.8	2.5
B-2	5,622,514	13	9.7	48	35.9	3.7
Subgroup total	7,512,494		11		39.7	
Students and exchange visitors						
F1	329,844	224	116.4	316	164.2	1.4
F2	21,729	199	6.8	260	8.9	1.3
J1	248,321	110	43.0	148	57.9	1.3
J2	29,213	196	9.0	253	11.6	1.3
M1	5,022	101	0.8	147	1.2	1.5
M2	602	29	0.0	85	0.1	2.9
Subgroup total	634,731		176.1		243.9	
Temp workers and families						
CW1	n/a	n/a	n/a	n/a	n/a	n/a
E1	36,489	60	2.2	114	4.1	1.9
E2	101,435	56	5.6	110	11.0	2.0
E3	n/a	n/a	n/a	n/a	n/a	n/a
H1B	262,880	91	23.7	173	45.0	1.9
H1B1	n/a	n/a	n/a	n/a	n/a	n/a
H1C	18	120	0.0	189	0.0	1.6
H2A	8,095	139	1.1	154	1.2	1.1
H2B	45,963	170	7.7	171	7.8	1.0

Visa type	No.† with visa type	Median LOS for visa type (days)	Weighted median LOS (days)	Mean LOS for visa type (days)	Weighted mean LOS (days)	Ratio, wt. mean to wt. median
H2R	n/a	n/a	n/a	n/a	n/a	n/a
H3	1,997	99	0.2	158	0.3	1.6
H4	70,597	198	13.8	268	18.7	1.4
I1	27,403	9	0.2	49	1.3	5.4
L1	247,850	37	9.1	83	20.4	2.2
L2	100,294	128	12.7	177	17.6	1.4
O1	20,469	30	0.6	76	1.5	2.5
O2	4,139	10	0.0	41	0.2	4.1
O3	2,618	98	0.3	166	0.4	1.7
P1	31,296	11	0.3	41	1.3	3.7
P2	1,463	21	0.0	82	0.1	3.9
P3	7,247	14	0.1	35	0.3	2.5
P4	1,065	109	0.1	144	0.2	1.3
Q1	1,250	164	0.2	194	0.2	1.2
R1	12,240	94	1.1	169	2.0	1.8
R2	2,716	110	0.3	179	0.5	1.6
TD	4,068	326	1.3	293	1.2	0.9
TN	18,983	311	5.8	246	4.6	0.8
Subgroup total	1,010,575		86.6		140.0	
Diplomat and other representatives						
A1 to A3	111,359	10	5.5	74	40.6	7.4
G1 to G5	82,450	32	13.0	80	32.5	2.5
N1 to N7	8,957	58	2.6	128	5.7	2.2
Subgroup total	202,766		21.1		78.8	
All other classes						
K1	3,889	161	1.5	220	2.0	1.4
K2	658	95	0.1	168	0.3	1.8
K3	1,546	98	0.4	136	0.5	1.4
K4	228	129	0.1	153	0.1	1.2
N8 to N9	52	39	0.0	105	0.0	2.7
T1 to T4	449	111	0.1	132	0.1	1.2
U1 to U4	134	114	0.0	153	0.0	1.3
V1 to V3	12,763	302	9.0	249	7.4	0.8
C1	168,222	1	0.4	8	3.1	8.0
C2	1,637	63	0.2	134	0.5	2.1
C3	8,496	1	0.0	4	0.1	4.0
C4	230,410	1	0.5	2	1.1	2.0
Q2 to Q3	373	148	0.1	172	0.1	1.2
Subgroup total	428,857		12.5		15.4	

Source: Grieco EM. Length of visit of nonimmigrants departing the United States in 2003: U.S. Department of Homeland Security, Office of Immigration Statistics; 2005.

*Groupings according to Table 28 of the 2009–2013 Yearbooks of Immigration Statistics published by the U.S. Department of Homeland Security, Office of Immigration Statistics.

† LOS is length of stay; No. is number, wt. is weighted

‡ Weighted LOS was calculated by dividing the number of a given visa type by the total for the visa group and multiplying by the mean or median LOS for that specific visa. The sum of the weighted LOS gave the weighted mean or median LOS for the visa subgroup.

Technical Appendix Table 2. Overall Calculation of 95% Confidence Interval for Average Annual Incident TB Cases in Study Population

Group	Best estimate, TB* cases	Lower bound	Upper bound	Width	1/2 Width	Proportion, 1/2 width to incident TB†
Subpopulation with authorization, 2010	2,631	2,309	2,956			
Subpopulation without authorization, 2010	222	165	298			
Total study population, 2010	2,853	2,474	3,254	780	390	0.137
Total study population, 5-year annual average	2,827	2,440‡	3,213§			

* TB is tuberculosis.

† Proportion used to calculate the overall study lower and upper bounds; TB is tuberculosis.

‡ The lower bound of the 95% confidence interval, calculated as [incident TB - (incident TB * proportion)].

§ The upper bound of the 95% confidence interval, calculated as [incident TB + (incident TB * proportion)].

Technical Appendix Table 3. Sensitivity Analysis of Admissions, Person-years, Incident Tuberculosis Cases, and Case Rates Stratified by Visa Group and Burden Level for Persons Temporarily in U.S. with Authorization†, 2008–2012

Visa group	Admissions (%)	PY* (%)	TB* No. cases (%)	TB* No./100,000 PY (95% CI)	TB* No./ 100,000 admissions (95% CI)
Tourist and business traveler	201,578,207 (25)	5,034,802 (29)	1,893 (29)	38 (32–43)	1 (1–1)
High burden countries	13,858,503 (2)	390,778 (2)	771 (12)	197 (168–227)	6 (5–6)
Medium burden countries	126,042,138 (15)	3,415,731 (20)	1,017 (16)	30 (25–34)	1 (1–1)
Low burden countries	61,677,566 (8)	1,228,294 (7)	105 (2)	9 (7–10)	0 (0–0)
Student/exchange visitor‡	9,417,888 (1)	4,543,757 (27)	2,653 (41)	58 (50–67)	28 (24–32)
High burden	1,862,032 (0)	898,357 (5)	1,473 (23)	164 (139–189)	79 (67–91)
Medium burden	4,932,913 (1)	2,379,935 (14)	1,095 (17)	46 (39–53)	22 (19–26)
Low burden	2,622,943 (0)	1,265,466 (7)	86 (1)	7 (6–8)	3 (3–4)
Temporary worker‡	12,904,847 (2)	3,063,082 (18)	1,435 (22)	47 (40–54)	11 (10–13)
High burden	2,154,566 (0)	511,406 (3)	993 (15)	194 (165–223)	46 (39–53)
Medium burden	5,252,984 (1)	1,246,843 (7)	363 (6)	29 (25–34)	7 (6–8)
Low burden	5,497,297 (1)	1,304,833 (8)	79 (1)	6 (5–7)	1 (1–2)
Diplomat and other representative‡	1,761,901 (0)	101,852 (1)	65 (1)	64 (54–73)	4 (3–4)
High burden	332,182 (0)	19,203 (0)	45 (1)	232 (198–267)	13 (11–16)
Medium burden	819,393 (0)	47,368 (0)	618 (0)	37 (31–42)	2 (2–3)
Low burden	610,326 (0)	35,282 (0)	3 (0)	8 (7–9)	0 (0–1)
All other classes	2,267,465 (0)	83,339 (1)	87 (1)	104 (89–120)	4 (3–4)
High burden	905,522 (0)	31,011 (0)	72 (1)	232 (197–267)	8 (7–9)
Medium burden	1,107,955 (0)	37,944 (0)	14 (0)	38 (32–44)	1 (1–2)
Low burden	253,988 (0)	14,385 (0)	1 (0)	5 (4–5)	0 (0–0)
Unknown visa class	1,123,438 (0)	21,313 (0)	12 (0)	57 (49–66)	1 (1–1)
High burden	71,316 (0)	1,563 (0)	4 (0)	236 (200–271)	5 (4–6)
Medium burden	792,676 (0)	17,374 (0)	8 (0)	47 (40–54)	1 (1–1)
Low burden	259,446 (0)	2,376 (0)	0 (0)	17 (14–20)	0 (0–0)
Canadian and Mexican nonimmigrant without an I-94	592,645,430 (72)	4,266,235 (14)	371 (3)	9 (7–10)	0 (0–0)
Total	821,699,176	17,114,381	6,516		
Annual average	164,339,835	3,422,876	1,303		

* CI is confidence interval; PY is person-years; TB is tuberculosis.
† High burden countries were defined as having ≥100 TB incident cases per 100,000 PY; medium-burden countries as having a case rate of 15–99, and low-burden countries, 0–14. Weighted median length of stay was used for all these groups, except those without an I-94 form, as only the mean was available.
‡ Corresponding spouses and children are also included in each of these categories; See Appendix Table 1 for full list of visas included in each subgroup.

Technical Appendix Table 4. Sensitivity Analysis for Estimated Incident Tuberculosis Cases for All Subgroups at Risk of Treatment Interruption Due to Leaving U.S.

Study subgroup	2008	2009	2010	2011	2012	Yearly average (%)
Resided in U.S. with authorization						
No.* cases, tourist & business traveler	337	305	374	430	446	378 (25)
No. cases, student & exchange visitor†	503	475	567	571	538	531 (34)
No. cases, temporary worker†	293	244	293	311	294	287 (19)
No. cases diplomat & other representative†	13	12	13	13	13	13 (1)
No. cases, all other NIV classes	19	18	17	17	16	17 (1)
No. cases, unknown NIV class	2	2	3	2	2	2 (0)
No. cases, Canadian non-I-94s	21	19	15	15	15	17 (1)
No. cases, Mexican non-I-94s	64	60	54	52	55	57 (4)
Resided in U.S. without authorization						
No. cases, detained then removed	173	175	166	196	218	186 (12)
No. cases, non-detained, removed	6	6	6	6	6	6 (0)
No. cases, MX voluntary departure	35	42	39	40	39	39 (3)
No. cases, all other voluntary departure	10	12	11	11	11	11 (1)
Total	1,476	1,370	1,558	1,664	1,653	1,544

* MX is Mexican; NIV is nonimmigrant visa; No. is number.
† Corresponding spouses and children were included in each of these categories; See Appendix Table 1 for full list of visas included in each subgroup.

Technical Appendix Table 5. Sensitivity Analysis for Estimated Incident Tuberculosis Cases Referred for Transnational Care Continuity Services

Year	Study total estimated cases*	CureTB-managed cases†	Health Network–managed cases
2008	1,476	90	106
2009	1,370	111	95
2010	1,558	108	109
2011	1,664	111	134
2012	1,653	90	155
Annual average (% referred)	1,544 (14)	102	120
Total incident TB cases from detained &removed subgroup (% referred)	928 (67)	180	442
Annual average for subgroup	186	36	88

*Study population was defined as nonimmigrants, nonrefugees who were born outside of the U.S., had active tuberculosis while in the U.S., and then left the U.S. before treatment completion was possible, 2008–2012, inclusive. TB cases estimates included use of weighted median lengths of stay.
† 2008 and 2009 numbers were extrapolated using previously reported data from 2010–2012 (*25, 26*).

Technical Appendix Table 6. Sensitivity Analysis for Overall Calculation of 95% Confidence Interval for Average Annual Incident TB Cases in Study Population

Group	Best estimate, TB* cases	Lower bound	Upper bound	Width	1/2 Width	Proportion, 1/2 width to incident TB†
Subpopulation with authorization, 2010‡	1,336	1,106	1,569			
Subpopulation without authorization, 2010	222	165	298			
Total study population, 2010	1,558	1,271	1,867	596	298	0.19
Total study population, 5-year annual average	1,544	1,249§	1,840¶			

* TB is tuberculosis.
† Proportion used to calculate the overall study lower and upper bounds; TB is tuberculosis.
‡ Estimates for incident TB cases included the use of both weighted mean and median lengths of stay.
§ The lower bound of the 95% confidence interval, calculated as [incident TB - (incident TB * proportion)].
¶ The upper bound of the 95% confidence interval, calculated as [incident TB + (incident TB * proportion)].
